# Anti-IL17A in Axial Spondyloarthritis—Where Are We At?

**DOI:** 10.3389/fmed.2017.00001

**Published:** 2017-01-18

**Authors:** Peter P. Cheung

**Affiliations:** ^1^Division of Rheumatology, National University Hospital, Singapore, Singapore; ^2^Yong Loo Lin School of Medicine, National University, Singapore, Singapore

**Keywords:** ankylosing spondylitis, biologics, IL17A, spondyloarthritis, treatment outcome

## Abstract

Knowledge regarding the mechanisms of the IL17–IL23 pathway and its role in spondyloarthritis (SpA) has been pivotal to the development of IL-17 blockade in patients with axial SpA. Previously, only anti-TNF has proven to be clinically efficacious in patients with active disease, despite non-steroidal anti-inflammatory drugs and physiotherapy. However, up to 50% fail to achieve a clinically significant response. Secukinumab, a fully humanized monoclonal antibody targeting IL-17A, has recently been approved for use in patients with active ankylosing spondylitis. Clinical studies and current issues surrounding the use of secukinumab will be discussed.

## Introduction

Ankylosing spondylitis (AS) is a chronic inflammatory disease predominantly affecting the axial skeleton and possibly the peripheral joints and entheses with a major impact on quality of life (1). It is the prototype of several inter-related inflammatory arthritides, referred to as spondyloarthritis (SpA), and grouped together as it shares a number of common genetic, pathogenic, and phenotypic features (1).

Non-steroidal anti-inflammatory drugs (NSAIDs) and physiotherapy including stretching exercises remain the initial first-line treatment for patients (2, 3). Disease-modifying anti-rheumatic drugs are not effective in patients with axial involvement and patients with continual disease activity, despite NSAIDs and physiotherapy would benefit from anti-TNF (4–7). Although dramatic improvements in disease activity and physical function are usually expected, up to 40–50% of these patients fail to have a clinically significant response (2, 3). Moreover, anti-TNF cannot maintain long-term remission, with most patients flaring after stopping the treatment (8). Anti-TNF can effectively control inflammation and therefore can prevent joint destruction (9). However, it does not halt new bone formation (10). This indicates other potentially important pathogenic pathways are involved in SpA.

Treatment goals are now focused on remission or achievement of low disease activity (11). The number of drugs available for patients with SpA that are formally available for clinical use is significantly less than other rheumatic diseases such as rheumatoid arthritis (RA). While there is proven efficacy in a number of biologic therapies for RA, studies evaluating tocilizumab, abatacept, and rituximab have been disappointing in SpA (12–15). There is increasing evidence that IL-17A blockade can be effective in patients with active SpA of which this review will focus on.

## Pathophysiology of SpA and IL17–IL23 Axis

Previously, the pathogenesis has centered on description of the Th1 pathway and TNF-alpha (16). Knowledge regarding the mechanisms of the IL17–IL23 pathway has increased with genetic, experimental models and functional data suggesting that it plays a crucial role in SpA (16, 17). First evidence came from genetic studies that showed protective polymorphisms in the IL-23 receptor gene (rs11209026, Arg381Gln) for AS (18) by impairing function of Th17 cells to produce IL-17 through the IL-23-dependent pathway. In addition, animal models have demonstrated that HLA-B27 misfolding and homodimer formation can trigger IL-23 and IL-17 production (19). Furthermore, upregulation of IL-17 expression due to expansion of Th17 cells is seen in HLA-B27 transgenic mice (20). Animal studies have also shown that IL-23 overexpression can induce a SpA-like disease with development of enthesitis (21) and bone formation (22).

The numbers of IL-17-producing cells are also elevated in the circulation and target tissues in AS patients (23), and the frequency of IL17+ CD4+ T cells is increased in early axial SpA patients, with or without MRI evidence of sacroiliitis (24). Moreover, macrophages of AS patients can produce higher levels of IL-23 with increased IL17-producing innate immune cells (25, 26).

## IL17 Inhibition in SpA—Clinical Studies

Due to previous preclinical data and its proven efficacy in psoriasis and psoriatic arthritis (27), a number of monoclonal antibodies have been developed to target IL-17A in SpA. IL-17A is the prototype in the IL-17 family of cytokines. To date, IL-17A and IL-17F are the most studied, although the latter is less implicated in autoimmunity, partly due to significantly reduced signaling triggered by IL-17F (28). Available agents so far include secukinumab (anti-IL17A), ixekizumab (anti-IL17A), and brodalumab (anti-IL17RA) for example (29). To date, only secukinumab is clinically approved, while the others are still under evaluation. Secukinumab is a fully anti-interleukin 17A monoclonal antibody, highly specific to the human immunoglobulin G1k (IgG1k) subclass. The current recommended dosing for axial SpA is weekly loading dose of 150 mg subcutaneously for 1 month, followed by monthly 150 mg of subcutaneous injections.

A Phase 2 open-label proof-of-concept study using secukinumab in 37 AS patients was conducted in Netherlands and Germany over 28 weeks (30). In this study, two intravenous doses of secukinumab 10 mg/kg, 3 weeks apart, was administered. Like other pivotal anti-TNF trials (4–7), the primary outcome was the proportion of patients achieving ASAS20 [Table 1; (31)] and was achieved in 59% of patients at 6 weeks.

**Table 1:** **ASAS response criteria for clinical trials (26)**.

Response criteria	Components included in the response criteria
ASAS20	At least 20% improvement from baseline and an absolute improvement from baseline of at least 1 U (on scale 0–10) in at least three out of four ASAS assessment domains, and no worsening >1 U in the remaining one out of the four domains Patient global assessment Spinal pain Physical function from BASFI Inflammation/morning stiffness
ASAS40	At least 40% improvement from baseline and an absolute improvement from baseline of at least 2 U (on scale 0–10) in at least three out of four ASAS assessment domains, and no worsening >2 U in the remaining one out of the four domains Patient global assessment Spinal pain Physical function from BASFI Inflammation/morning stiffness
ASAS partial remission	A score of <2 in each of the four ASAS assessment domains

*ASAS, Assessment of Spondyloarthritis International Society; BASFI, Bath Ankylosing Spondylitis Functional Index*.

This paved way to Phase 3 clinical studies of secukinumab in AS (MEASURE 1, *n* = 371 and MEASURE 2, *n* = 219) (32). Unlike the pivotal anti-TNF trials (4–7), not all of the patients were anti-TNF naïve (only 71 and 61% of patients were anti-TNF naïve in MEASURE 1 and MEASURE 2, respectively).

On the one hand, in MEASURE 1, patients received intravenous loading doses (10 mg/kg) at 0, 2, and 4 weeks, followed by either monthly subcutaneous secukinumab, at doses of 150 or 75 mg, vs. placebo. The primary outcome (ASAS20) at Week 16 was achieved by 61% in the 150 mg arm and 60% in the 75 mg arm. ASAS40 was achieved by 49 and 34%, respectively, while ASAS partial remission was 15 and 16%, respectively. On the other hand, in MEASURE 2, patients received weekly subcutaneous loading doses of either 150 or 75 mg for 1 month followed by monthly administration vs. placebo. ASAS20 response at Week 16 was achieved in 61% of patients receiving the 150 mg compared to 41% in those receiving 75 mg. ASAS40 response rates were 43 and 31%, respectively, while partial remission was achieved in 14 and 15%, respectively. On the basis of superior outcomes in majority of the domains in the 150 mg dosing, this was the recommended dosing for SpA. One reasonable explanation to the better outcomes at 75 mg dosing in MEASURE 1 compared to MEASURE 2 study was due to the initial intravenous loading dose received by patients in MEASURE 1 (32).

### Anti-TNF Naïve vs. TNF Non-Responders

**Figure 1:**
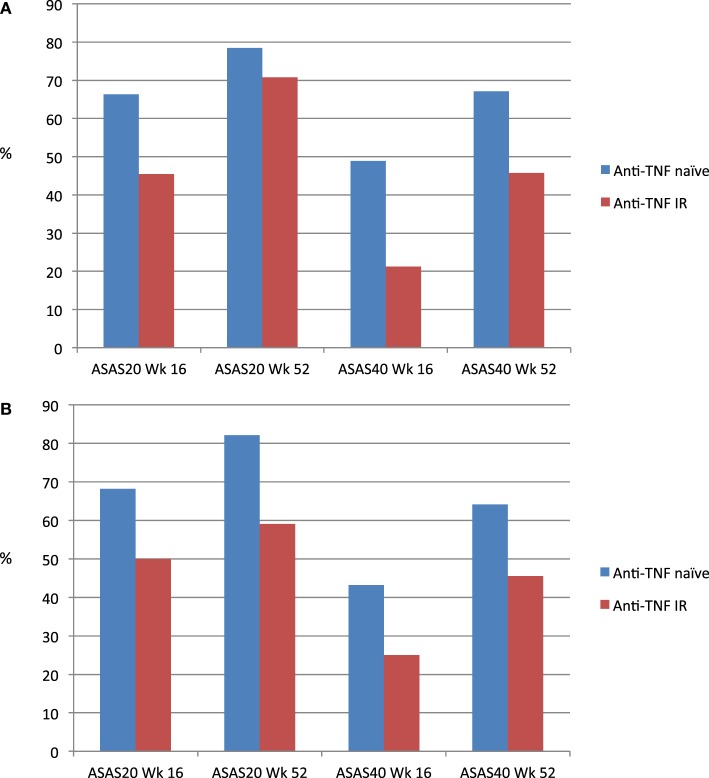
**(A)** ASAS20 and ASAS40 response rates (MEASURE 1) for secukinumab 150 mg dosing. **(B)** ASAS20 and ASAS40 response rates (MEASURE 2) for secukinumab 150 mg dosing.

As mentioned previously, around 30% were not anti-TNF naïve. Although inclusion criteria such as disease activity and failure of first-line therapy with NSAIDs were comparable to pivotal anti-TNF trials (4–7), patients with spinal ankylosis were excluded in both MEASURE 1 and MEASURE 2. At Week 16, response rates for anti-TNF were higher than anti-TNF inadequate responders in MEASURE 1 (Figure 1A) and MEASURE 2 (Figure 1B) (32). However, up to 60% of anti-TNF inadequate responders had also achieved ASAS20 response by 52 weeks (33).

### Extra-Articular Manifestations

Although there is good evidence of its efficacy in both axial and peripheral musculoskeletal manifestations, as well as extra-articular manifestations such as psoriasis (24), the effectiveness on uveitis is less clear. To date, secukinumab is not efficacious in patients with inflammatory bowel disease (34), and it is unclear if it would induce the condition. However, studies of Phase 2 and Phase 3 clinical trials of psoriasis, psoriatic arthritis, and AS patients who received secukinumab indicated that inflammatory bowel disease incidences or flares were infrequently reported (35).

### Efficacy of Secukinumab at 2 Years

Recently, longer term 2-year data were available, demonstrating that secukinumab provided sustained improvements in signs and symptoms of AS, with improved physical function regardless of anti-TNF status (36). In MEASURE 2, both 75 and 150-mg dosing achieved similar ASAS20 and ASAS40. The proportion of patients in both doses that achieved ASAS20 and ASAS40 was 72 and 48%, respectively, by 2 years. In those that continued the trial, the proportion of anti-TNF inadequate responders and anti-TNF naïve patients that achieved ASAS20 and ASAS40 response were similar at 2 years as well. Similarly, sustained improvements through 2 years including disease activity, pain, physical function, and fatigue as well as general and AS-specific measures of quality of life were demonstrated (37).

### Radiographic Progression

To date, radiographic data are only available in SpA patients with radiographic sacroiliitis, up to 2 years for secukinumab (38–40), which are short to evaluate radiographic progression since the process is slow. In MEASURE 1, both 150 and 75 mg doses were pooled together as there were no major clinical differences. Of the 371 patients, 80% of patients showed no radiographic progression [modified stoke ankylosing spondylitis spine score (mSASSS) change ≤0] in patients receiving secukinumab over 104 weeks (40). Factors that contributed to progression were similar to anti-TNF studies such as males, baseline syndesmophytes, and elevated CRP levels at baseline (40). The authors commented that changes in mSASSS through 2 years may be lower than those reported in earlier observational and interventional studies in AS, but the question whether this therapy may reduce bone formation remains (40). In fact, the controversy of bone formation from anti-TNF in axial SpA has not been fully resolved. While earlier studies on adalimumab, infliximab, and etanercept failed to demonstrate reduction in radiographic progression in axial SpA (10, 41, 42), further studies have indicated that radiographic progression could be delayed if anti-TNF was given early, or for a long period of time, presumably because the inflammation specifically causing bone formation has been suppressed (43, 44). Although the proportion of patients who had radiographic progression in MEASURE 1 was lower than the anti-TNF studies (10, 41, 42), there was no control arm. Only head-to-head studies evaluating anti-TNF and secukinumab can answer this question. Moreover, there is a need at least to compare secukinumab with a historical control as well as having longer term studies to answer this question.

### Safety Information

As expected, patients who received secukinumab had more infections compared to placebo (30 vs. 12% in MEASURE 1 and 32 vs. 27% in MEASURE 2) (32). Exposure adjusted incidence rates of serious adverse events were 73.5 and 59.4 events per 100 patient-years among patients receiving 150 and 75 mg, respectively, in MEASURE 1, compared to 60.5 and 89.1, respectively, for MEASURE 2. The most common side effect is nasopharyngitis followed by headaches. *Candida* infection, which could theoretically increase with IL-17A inhibition, was reported in three cases, with pooled adjusted incidence of candidiasis in secukinumab-treated patients across the two studies of 0.9 events per 100 patient-years of exposure. Crohn’s disease was an adverse event in five patients, while uveitis was reported in five patients vs. two patients receiving placebo. Major adverse cardiac events for the entire treatment period of both studies were 0.4 per 100 patient-years, consistent with incidence rates in other SpA (32). Of the results so far, there is no increased reactivation or cases of tuberculosis. No reports of increased suicidality were seen in those treated with secukinumab, which was observed previously in trials of brodalumab in psoriasis patients. Four malignant or unspecified tumors were reported. Immunogenicity was also low with no antidrug antibodies detected (32).

## Issues of IL17 Inhibition

A number of issues remain unresolved. There are no data on the efficacy of secukinumab in non-radiographic SpA. It is also unclear whether a loading dose of weekly 150 mg subcutaneous injections is necessary. Although secukinumab is recommended at dose of 150 mg, it would be interesting to characterize patients that would respond to 75 mg and to evaluate the clinical outcomes between the two dosages beyond 2 years. More evaluation on patients who did not respond to anti-TNF would be necessary to establish if secukinumab is superior to switching class of anti-TNF (45). Logically, from our experience in RA, we would expect switching to a different class of medication to be more efficacious.

In addition, reactivation of TB should be monitored, since anti-TNF poses this potential risk. So far, *in vitro* testing with peripheral blood mononuclear cells infected with *Mycobacterium tuberculosis* treated with anti-TNF and IL-17A inhibition showed that anti-IL-17A treatment *in vitro* did not reverse *M. tuberculosis* dormancy in a human granuloma model (46). Long-term clinical data would be necessary to prove this theoretical advantage. Specifically for areas such as Asia, where TB is common, this question is important to evaluate.

Like that of anti-TNF, the debate about whether co-medications are necessary, even in cases of peripheral predominant disease, should be evaluated. Although present data show very little development of anti-secukinumab antibodies, it remains to be seen whether patients will develop secondary non-response; however, the data currently show low immunogenicity rates. It is also unknown whether secukinumab would be effective in patients with spinal ankylosis. It is also unclear how long it takes for patients to flare after stopping secukinumab, an observation already well known with anti-TNF (8).

Finally, structural progression in AS, characterized mainly by new bone formation, can lead to ankylosis of the spine. The question whether secukinumab can halt bone formation remains unanswered.

## Current Ongoing Studies

Ongoing studies are evaluating some of these issues. The 5-year data for MEASURE 2 should be available in the future. Another study is evaluating the optimal dosing of secukinumab by comparing dosing at 300 vs. 150 mg (http://ClinTrials.gov number NCT02008916) each receiving loading dose 10 mg/kg initially. This is considering that the current indicated dosing for psoriasis and those with psoriatic arthritis who were anti-TNF non-responders is recommended at 300 mg. Another study will evaluate the need for initial loading dose (NCT02159053). Non-radiographic axial SpA with or without loading dose 150 mg is currently being evaluated (NCT02696031). Finally, the effects of NSAID tapering in patients with secukinumab (NCT02763046) will also be studied.

## Other Promising Therapies

Other therapy that is important to consider is that of ustekinumab (47) and tofacitinib (48). Promising results for tofacitinib was recently presented, with Phase 2 results showing significant difference compared to placebo in terms of disease activity, function, and mobility (48).

## Practical Applications on Which Therapy to Use—Anti-TNF or IL-17A Blockade?

At present, anti-TNF is still the preferred first-line biologic agent for patients with active axial SpA, and either switching to another anti-TNF or to IL-17A blockade is equally acceptable if there is primary or secondary non-response. However, there are instances where IL-17A may be preferred, for example, in patients with higher risk of tuberculosis or in cases with psoriasis with high burden of peripheral arthritis, although extensive psoriasis is not routinely seen in patients with axial SpA. In cases of history of inflammatory bowel disease, it would be preferable to avoid using secukinumab, although more information in the future is required.

## Conclusion

It is currently an exciting time for biologic therapies available in SpA. Secukinumab has now emerged as an alternative therapy for patients with SpA. Despite a number of unanswered questions still, current preliminary results appear promising.

## Author Contributions

I declare this is my original work, and I am the principal author of this manuscript.

## Conflict of Interest Statement

The author declares that the research was conducted in the absence of any commercial or financial relationships that could be construed as a potential conflict of interest.

## References

[1] Dougados M Baeten D. Spondyloarthritis. Lancet (2011) 377:2127–37. 10.1016/S0140-6736(11)60071-8 21684383

[2] Braun J van den Berg R Baraliakos X Boehm H Burgos-Vargas R Collantes-Estevez E 2010 update of the ASAS/EULAR recommendations for the management of ankylosing spondylitis. Ann Rheum Dis (2011) 70:896–904. 10.1136/ard.2011.151027 21540199 PMC3086052

[3] Ward MM Deodhar A Akl EA Lui A Ermann J Gensler LS American college of rheumatology/spondylitis association of America/spondyloarthritis research and treatment network 2015 recommendations for the treatment of ankylosing spondyloarthritis. Arthritis Rheumatol (2016) 68:282–98. 10.1002/art.39298 26401991 PMC5123840

[4] Inman RD Davis JC Jr van der Heidje D Diekman L Sieper J Kim S Efficacy and safety of golimumab in patients with ankylosing spondylitis: results of a randomized, double-blind, placebo-controlled, phase III trial. Arthritis Rheum (2008) 58:3402–12. 10.1002/art.23969 18975305

[5] Van der Heijde D Dijkmans B Geusens P Sieper J Dewoody L Williamson P Efficacy and safety of infliximab in patients with ankylosing spondylitis: results of a randomized, placebo controlled trial (ASSERT). Arthritis Rheum (2005) 52:582–91. 10.1002/art.20985 15692973

[6] Van der Heijde D Kivitz A Schiff MH Sieper J Dijkmans B Braun J Efficacy and safety of adalimumab in patients with ankylosing spondylitis: results of a multicenter, randomized, double blind, placebo-controlled trial. Arthritis Rheum (2006) 54:2136–46. 10.1002/art.21913 16802350

[7] Davis JC Jr van der Heijde D Braun J Dougados M Cush J Clegg DO Recombinant human tumor necrosis factor receptor (etanercept) for treating ankylosing spondylitis: a randomized controlled trial. Arthritis Rheum (2003) 48:3230–6. 10.1002/art.11325 14613288

[8] Baraliakos X Kiltz U Heldmann F Sieper J Braun J. Withdrawal of biologic therapy in axial spondyloarthritis: the experience in established disease. Clin Exp Rheumatol (2013) 31(Suppl 78):S43–6. 24129136

[9] Van der Heijde D Kavanaugh A Gladman DD Antoni C Krueger GG Guzzo C Infliximab inhibits progression of radiographic damage in patients with active psoriatic arthritis through one year of treatment: results from the induction and maintenance psoriatic arthritis clinical trial. Arthritis Rheum (2007) 56:2698–707. 10.1002/art.22805 17665424

[10] van der Heijde D Landewé R Baraliakos X Houben H van Tubergen A Williamson P Radiographic findings following two years of infliximab therapy in patients with ankylosing spondylitis. Arthritis Rheum (2008) 58:3063–70. 10.1002/art.23901 18821688

[11] Smolen JS Braun J Dougados M Emery P FitzGerald O Helliwell P Treating spondyloarthritis, including ankylosing spondylitis and psoriatic arthritis, to target: recommendations of an international task force. Ann Rheum Dis (2014) 73:6–16. 10.1136/annrheumdis-2013-203419 23749611 PMC3888616

[12] Sieper J Braun J Kay J Badalamenti S Radin AR Jiao L Sarilumab for the treatment of ankylosing spondylitis: results of a phase II, randomized, double-blind, placebo-controlled study (ALIGN). Ann Rheum Dis (2014) 74:1051–7. 10.1136/annrheumdis-2013-204963 24550171 PMC4431338

[13] Sieper J Porter-Brown B Thompson L Harari O Dougados M Assessment of short-term symptomatic efficacy of tocilizumab in ankylosing spondylitis: results of a randomized, placebo-controlled trials. Ann Rheum Dis (2014) 73:95–100. 10.1136/annrheumdis-2013-203559 23765873 PMC3888605

[14] Song IH Heldmann F Rudwaleit M Haibel H Weiss A Braun J Treatment of active ankylosing spondylitis with abatacept: an open-label, 24-week pilot study. Ann Rheum Dis (2011) 70:1108–10. 10.1136/ard.2010.145946 21415053

[15] Song IH Heldmann F Rudwaleit M Listing J Appel H Braun J Different response to rituximab in tumor necrosis factor blocker naïve patients with active ankylosing spondylitis and in patients in whom tumor necrosis factor blockers have failed: a twenty four week clinical trial. Arthritis Rheum (2010) 62:1290–7. 10.1002/art.27383 20461780

[16] Paine A Ritchlin CT. Targeting the interleukin-23/17 axis in axial spondyloarthritis. Curr Opin Rheumatol (2016) 28:359–67. 10.1097/BOR.0000000000000301 27152702 PMC5777167

[17] Hreggvidsdottir HS Noordenbos T Baeten DL Inflammatory pathways in spondyloarthritis. Mol Immunol (2014) 57:28–37. 10.1016/j.molimm.2013.07.016 23969080

[18] Wellcome Trust Case Control Consortium, Australo-Anglo-American Spondylitis Consortium (TASC) Burton PR Clayton DG Cardon LR Craddock N Association scan of 14500 nonsynonymous SNPs in four diseases identifies autoimmunity variants. Nat Genet (2007) 39:1329–37. 10.1038/ng.2007.17 17952073 PMC2680141

[19] Colbert RA Turner MJ DeLay ML Smith JA Klenk EL Sowders DP HLA-B27 misfolding activates the IL-23/IL-17 axis via the unfolded protein response in transgenic rats: evidence for a novel mechanism of inflammation. Arthritis Rheum (2007) 54:S515.

[20] Glatigny S Fert I Blaton MA Lories RJ Araujo LM Chiocchia G Proinflammatory Th17 cells are expanded and induced by dendritic cells in spondyloarthritis-prone HLA-B27-transgenic rats. Arthritis Rheum (2011) 64:110–20. 10.1002/art.33321 21905004

[21] Abe Y Ohtsuji M Ohtsuji N Lin Q Tsurui H Nakae S Ankylosing enthesitis associated with upregulated IFN-gamma and IL-17 production in (BXSBxNZB) F(1) male mice: a new mouse model. Mod Rheumatol (2009) 19:316–22. 10.3109/s10165-009-0166-0 19357807

[22] Sherlock JP Joyce-Shaikh B Turner SP Chao CC Sathe M Grein J IL-23 induces spondyloarthropathy by acting on ROR-gammat+CD3+CD4-CD8-entheseal resident T cells. Nat Med (2012) 18:1069–76. 10.1038/nm.2817 22772566

[23] Appel H Maier R Wu P Scheer R Hempfing A Kayser R Analysis of IL-17 cells in facet joints of patients with spondyloarthritis suggests that the innate immune pathway might be of greater relevance than the Th17-mediated adaptive immune response. Arthritis Res Ther (2011) 13:R95 10.1186/ar3370 21689402 PMC3218910

[24] Jansen DT Hameetman M van Bergen J Huizinga TW van der Heijde D Toes RE IL-17 producing CD4+ T Cells are increased in early, active axial spondyloarthritis including patients without imaging abnormalities. Rheumatology (Oxford) (2015) 54:728–35. 10.1093/rheumatology/keu382 25288779

[25] Noordenbos T Yeremenko N Gofita T van de Sande M Tak PP Caňete JD Interleukin-17 positive mast cells contribute to synovial inflammation in spondyloarthritis. Arthritis Rheum (2012) 64:99–109. 10.1002/art.33396 21968742

[26] Shen H Goodall JC Hill Gaston JS. Frequency and phenotype of peripheral blood Th17 cells in ankylosing spondylitis and rheumatoid arthritis. Arthritis Rheum (2009) 60:1647–56. 10.1002/art.24568 19479869

[27] Mease PJ McInnes IB Kirkham B Kavanaigh A Rahman P van der Heijde D Secukinumab inhibition of interleukin-17A in patients with psoriatic arthritis. N Engl J Med (2015) 373:1329–39. 10.1056/NEJMoa1412679 26422723

[28] Gaffen SL. Structure and signalling in the IL-17 receptor family. Nature Rev Immunol (2009) 9:556–67. 10.1038/nri2586 19575028 PMC2821718

[29] Braun J Baraliakos X Kiltz U Secukinumab (AIN457) in the treatment of ankylosing spondylitis. Expert Opin Biol Ther (2016) 16:5711–22. 10.1517/14712598.2016.1167183 26982813

[30] Baeten D Baraliakos X Braun J Sieper J Emery P van der Heijde D Anti-interleukin-17A monoclonal antibody secukinumab in treatment of ankylosing spondylitis: a randomized, double-blind, placebo-controlled trial. Lancet (2013) 382:1705–13. 10.1016/S0140-6736(13)61134-4 24035250

[31] Sieper J Rudwaleit M Baraliakos X Brandt J Braun J Burgos-Vargas R The Assessment of spondyloarthritis international society (ASAS) handbook: a guide to assess spondyloarthritis. Ann Rheum Dis (2009) 68(Suppl 2):ii1–44. 10.1136/ard.2008.104018 19433414

[32] Baeten D Sieper J Braun J Baraliakos X Dougados M Emery P Secukinumab, an interleukin-17A inhibitor in ankylosing spondylitis. N Engl J Med (2015) 373:2534–48. 10.1056/NEJMoa1505066 26699169

[33] Baeten D Blanco R Geusens P Sieper J Jui-Cheng T Martin R Secukinumab provides sustained improvements in the signs and symptoms of active ankylosing spondylitis in anti-TNF naïve patients and those previously exposed to anti-TNF therapy: 52 week results from 2 randomised, double blind, placebo-controlled phase 3 trials [abstract]. Arthritis Rheumatol (2015) 67(Suppl 10).

[34] Hueber W Sands BE Lewitzky S Vandemeulebroecke M Reinisch W Higgins PD Secukinumab, a human anti-IL17A monoclonal antibody, for moderate to severe Crohn’s disease: unexpected results of a randomized, double-blind placebo controlled trial. Gut (2012) 61:1693–700. 10.1136/gutjnl-2011-301668 22595313 PMC4902107

[35] Schrieber S Sands BE Deodhar A Baeten D Huang J Gandhi K No increased incidence of inflammatory bowel disease among secukinumab treated patients with moderate to severe psoriasis, psoriatic arthritis or ankylosing spondylitis: data from 14 phase 2 and phase 3 clinical studies [abstract]. Ann Rheum Dis (2016) 75(Suppl 2):97.

[36] Marzo-Ortega H Legerton CW Sieper J Kivitz A Blanco R Cohen M Secukinumab provides sustained improvements in the signs and symptoms of active ankylosing spondylitis: 2 year results from a phase 3 trial with subcutaneous loading and maintenance dosing (MEASURE 2) [abstract]. Ann Rheum Dis (2016) 75(Suppl 2):812 10.1136/annrheumdis-2016-eular.2306

[37] Emery P Baeten D Deodhar A Wei A Geusens P Talloczy Z Secukinumab improves physical function and quality of life in patients with active ankylosing spondylitis: 2 year data from MEASURE 1, a phase 3 randomised trial [abstract]. Ann Rheum Dis (2016) 75(Suppl 2):818 10.1136/annrheumdis-2016-eular.2308

[38] Baraliakos X Borah B Braun J Baeten D Laurent D Sieper J Long-term effects of secukinumab on MRI findings in relation to clinical efficacy in subjects with active ankylosing spondylitis: an observational study. Ann Rheum Dis (2016) 75:408–12. 10.1136/annrheumdis-2015-207544 26248638

[39] Baraliakos X Deodhar AA Braun J Baeten D Dougados M Sieper J Effect of interleukin-17A inhibition on spinal radiographic changes through 2 years in patients with active ankylosing spondylitis: results of a phase 3 study with secukinumab [abstract]. Arthritis Rheumatol (2015) 67(Suppl):10.

[40] Braun J Baraliakos X Deodhar A Baeten D Sieper J Emery P Effect of secukinumab, an interleukin-17A inhibitor, on spinal radiographic changes through 2 years in patients with active ankylosing spondylitis: results of the phase 3 study, MEASURE 1. Ann Rheum Dis (2016) 75(Suppl 2):52 10.1136/annrheumdis-2016-eular.317725873634

[41] Van der Heijde D Landewe R Einstein S Ory P Vosse D Ni L Radiographic progression of ankylosing spondylitis after up to two years of treatment with etanercept. Arthritis Rheum (2008) 58:1324–31. 10.1002/art.23471 18438853

[42] Van der Heijde D Salonen D Weissman BN Landewe R Maksymowych WP Kupper J Assessment of radiographic progression in the spines of patients with ankylosing spondylitis treated with adalimumab for up to 2 years. Arthritis Res Ther (2009) 11:R127. 10.1186/ar2794 19703304 PMC2745811

[43] Haroon N Inman RD Learch TJ Weisman MH Lee M Rahbar MH The impact of tumor necrosis factor alpha inhibitors on radiographic progression in ankylosing spondylitis. Arthritis Rheum (2013) 65:2645–54. 10.1002/art.38070 23818109 PMC3974160

[44] Maas F Arends S Brouwer E Essers I van der Veer E Efde M Reduction in spinal radiographic progression in ankylosing spondylitis patients receiving prolonged treatment with TNF inhibitors. Arthritis Care Res (Hoboken) (2016). 10.1002/acr.23097 27696754

[45] Pradeep DJ Keat AC Gaffney K Brooksby A Leeder J Harris C Switching anti-TNF therapy in ankylosing spondylitis. Rheumatology (Oxford) (2008) 47:1726–7. 10.1093/rheumatology/ken334 18782856

[46] Kapoor N Kammuller M Kolattukudy PE No reactivation of dormant *Mycobacterium tuberculosis* in human in vitro granuloma model after anti-IL-17A treatment, in contrast to anti-TNF treatment [abstract]. Ann Rheum Dis (2016) 75(Suppl 2):434 10.1136/annrheumdis-2016-eular.3311

[47] Poddubnyy D Hermann KG Callhoff J Listing J Sieper J Ustekinumab for the treatment of patients with active ankylosing spondylitis: results of a 28 week prospective, open-label, proof of concept study (TOPAS). Ann Rheum Dis (2014) 74:817–23. 10.1136/annrheumdis-2013-204248 24389297

[48] Van der Heijde D Deodhar A Wei JC Drescher E Fleishaker D Hendrikx T Tofacitinib in patients with ankylosing spondylitis: a phase 2, 16 week randomized, placebo controlled, dose ranging study [abstract]. Ann Rheum Dis (2016) 75(Suppl 2):52 10.1136/annrheumdis-2016-eular.1847 28130206 PMC5738601

